# Precision medicine directed therapy enabling long-term survival in medulloblastoma: a case report

**DOI:** 10.3389/fonc.2026.1855992

**Published:** 2026-07-14

**Authors:** Laura Vazquez, Jonathan Lerch, Divya Gandra, Abigail Moore, Jessica Lincoln, Abhinav B. Nagulapally, Suzanne Treadway, Krishnamoorthy Thamburaj, Valerie Brown, Giselle Saulnier Sholler

**Affiliations:** 1 Pediatrics, Penn State College of Medicine, Hershey, PA, United States; 2 Pediatrics, Penn State Health Golisano Children’s Hospital, Hershey, PA, United States; 3 General Diagnostic Radiology, Penn State Health Golisano Children’s Hospital, Hershey, PA, United States

**Keywords:** case report, medulloblastoma, molecularly-guided, precision, Shh

## Abstract

Subgroup-SHH of medulloblastoma (MB) is primarily found in children, with an increased propensity for metastatic presentation that contributes to its poor prognosis. Current therapy approaches may include surgery, radiation, systematic chemotherapy, and autologous stem cell transplant (ASCT). At relapse, patients may receive additional chemotherapy and radiation if possible. The outcome for relapsed patients is 10-30% long term survival. Herein we present a case of a 6-year-old male with a pathologically confirmed diagnosis of subgroup-SHH MB, who was treated with upfront surgical resections, high-dose chemotherapy, and ASCT. Nine months after completion of upfront therapy, the subject relapsed. Subsequently, he was enrolled onto NMTRC009 (NCT02162732) and underwent biopsy with genomic sequencing (DNA whole exome and RNA transcriptome). A molecular tumor board, using precision medicine analysis, recommended vorinostat, vismodegib, curcumin, and palbociclib as targeted therapy. This therapy resulted in a complete tumor response and long-term survival. A cell line was derived from the subject’s tumor and used for *in vitro* testing. Combined molecular therapies demonstrated synergistic efficacy compared with single-agent treatment.

## 1 Introduction

Medulloblastoma (MB) is the most common pediatric malignant brain tumor, occurring in the cerebellum in the posterior fossa region. Accounting for 25% of all childhood brain tumors, diagnosis is most common between ages 5 and 9 years old with an annual incidence of 400 cases in the United States (1, 2). An estimated 30% of children diagnosed with MB will relapse, with a 10-30% 3-year overall survival (1, 3). Medulloblastoma may metastasize through the cerebrospinal fluid, leading to spread along the leptomeninges of the brain and spinal cord. MB is classified into four main subgroups: wingless (WNT), sonic hedgehog (SHH), non-WNT/non-SHH groups 3 and 4 (4–6). Each group is defined and clinically treated based on their implicated pathways and pathology (7).

The four primary subgroups are further delineated into 12 subtypes to distinguish the clinical and cytogenetic prognostic features (8). Inferior outcomes are associated with GLI2 and MYCN amplification, causing tumor suppression failure with activation of the SHH pathway. Additionally, those with TP53 mutations have greatly diminished outcomes in MB subgroup SHH compared to the WNT subgroup. Each is associated with increased metastasis, contributing to poor survival (8). The most common form of treatment is surgical removal of the tumor followed by radiation and chemotherapy. Recent findings have resulted in the development of molecular-based therapies that target specific genetic mutations in such tumors with the goal of improving survival outcomes and post-treatment quality of life (9, 10). This leads to the possibility of a precision medicine approach in medulloblastoma, in which treatment strategies are tailored to an individual’s tumor’s molecular profile to optimize efficacy while minimizing toxicity.

The HEADSTART III upfront protocol that this subject received reported five-year irradiation-free event free survival (EFS) of 78 ± 8% for Nodular/desmoplastic MB (11). Currently, there is no standard treatment for relapsed MB, and the literature is limited by the heterogeneity of patient population and treatments to provide adequate recommendations (12). The variability of treatments for refractory and relapsed MB often results in increased toxicity due to exposure to conventional chemotherapeutic agents in children and contributes to severe therapy-induced morbidities (2). Surgery provides a marginal increase in survival for relapsed patients, particularly if the relapse is localized (13).

## 2 Case description

A 6-year-old male presented with daily headaches and vomiting. Imaging workup showed multi-loculated left cerebellar and right temporal cystic lesions. No disease was detected in the spine or CSF. The subject underwent gross total resection of the left cerebellar region and near total resection of the right temporal lesion. Pathology revealed nodular desmoplastic MB and was positive for SHH pathway, TP53-wildtype, and negative for monosomy 6, MYCN or MYC gene amplification. The subject was considered Grade IV per the World Health Organization (WHO) tumor grading system, supported by the presence of a PTCH1 mutation and immunohistochemical findings (cytoplasmic β catenin without nuclear accumulation, nuclear/cytoplasmic YAP expression, and cytoplasmic GAB1 expression), consistent with SHH activation. The subject received alternating cycles of cisplatin, vincristine, cyclophosphamide, etoposide, and methotrexate (cycles 1, 3), temozolomide, etoposide, vincristine, and cyclophosphamide (cycle 2), temozolomide, etoposide, and cyclophosphamide (cycle 4), and cisplatin, cyclophosphamide, etoposide, and methotrexate (cycle 5). One cycle of high dose consolidation therapy, consisting of carboplatin, etoposide, and thiotepa, and ASCT, was given after completing cycle 5 of chemotherapy.

Nine months post completion of therapy, an MRI of the brain and spine showed disease recurrence in supra and infra-tentorial surgical beds, cerebellar vermis, thoracic and lumbar spine. At the time of this recurrence, the subject was asymptomatic. The right temporal tumor was resected, and pathology classified the lesion as recurrent MB, WHO Grade IV which matched initial diagnosis with SHH pathway and TP53-wildtype.

The subject was enrolled on the Beat Childhood Cancer Research Consortium (BCC) Clinical Trial “Molecular-guided therapy for the treatment of patients with relapsed refractory childhood cancers” (NCT02162732;NMTRC009) (14). The tumor was sent for DNA exome and RNA transcriptome sequencing, and the genomic analysis was discussed at a Molecular Tumor Board (MTB).

## 3 Diagnostic assessment

### 3.1 DNA-sequencing and RNA transcriptome analysis

Ashion’s Strexome assay, which included tumor-normal whole exome and tumor mRNA sequencing (RNASeq), was performed on the Illumina HiSeq 2500 Sequencer (PMID:34610968). Variant calls were used in the variant-drug matching algorithm for MTB discussion. Seurat was used for calling somatic single nucleotide variants and small indels, a custom copy number tool (tCoNuT) was used to call focal copy number events, Manta was used for structural variant calling and TopHat fusion was used for fusion detection. Exome Copy Number Variations (CNVs) were used to call chromosome arm-level events, filtering for regions containing >50 genes with a log2 ratio of tumor to matched normal of >=0.5 (gain) or <=-0.5 (loss). Chromosome arm-level events were called if gain or loss covered >=50% of arm.

**Figure 1:**
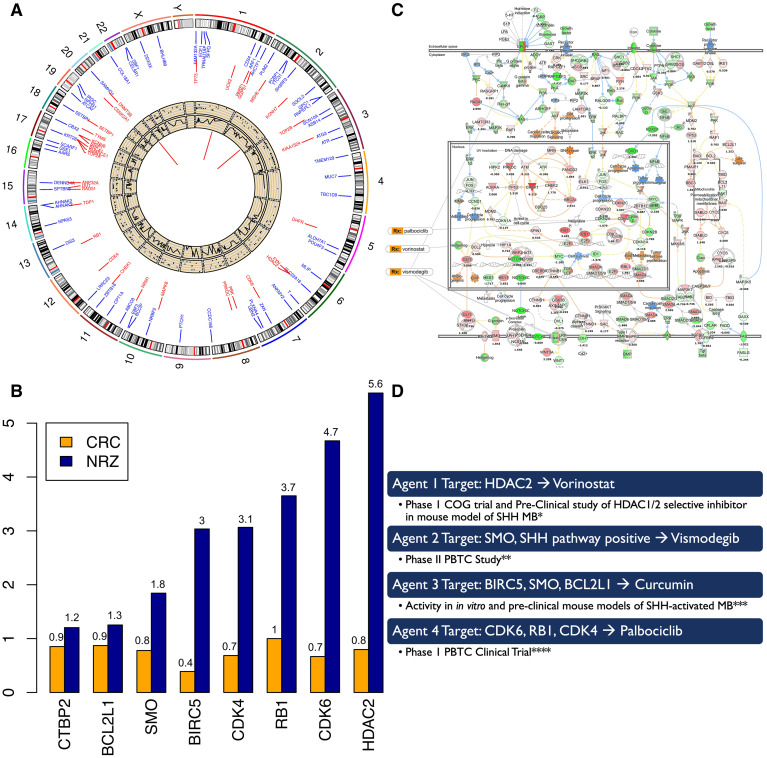
Genomic analysis of the MB subject. **(A)** Circos plot of WES DNA point mutation (blue) and RNASeq overexpression (orange) results. **(B)** Differential gene expression in the MB subject. Gene expression levels were compared to a normal whole-body reference composed of normal tissues and assigned as Z-score (NRZ) and tumor reference composed of childhood tumors and assigned as cumulative distribution-based statistic (CRC). **(C)** Activation of cancer pathway using IPA with expression scores and drug targets. **(D)** Supporting literature for rationale for choosing therapeutic regimen. *Fouladi et al, JCO 2010, 28:3623, **Coni, et al., scientific reports, 2017, 7:44079, ***Robinson et al, JCO 2015, 24:2646, ****Lee, et al., BMC cancer, 2011, 11:144, ****Elamin, et al., molecular carcinogenesis, 2010, 49:302.

DNA Mutation Analysis showed that the overall tumor mutation burden was low 1.87 Mut/Mb (**Figure 1A**, **Supplementary Table 1**) and microsatellite analysis was stable. Six somatic variants relevant to cancer were identified. Sixty-one SSID’s predicted to alter protein sequence at >1% variant allele frequency were identified (**Supplementary Table 1**). CNVs seen in several segmental changes consistent with chromosomal instability were identified using whole exome sequencing (WES). This includes large-scale gains in chromosomes 9 (9p13.1-24.3) and 1 (1q21.1-44), large-scale loss in chromosome 9 (9q21.11-34.3), as well as focal deletions of NOTCH1 and RALGS in 9q region (**Figure 1A**).

For gene expression analysis, sequence read processing included read trimming with Trimmomatic-0.36) (15), alignment with STAR 2.5.3 to GRCh37 (16) read counts using R package GenomicAlignments (17), regularized logarithm (rlog) values using DESeq2 (18). A previously published standardized Normal Reference (NRZ) Z−score method was used to determine statistically significant differences in each sample, relative to a whole−body reference of 22 normal tissue gene expression levels (19). Each tumor gene expression profile was also compared to expression profiles of 66 other childhood tumors, based on the cumulative distribution-based statistic (CRC) (**Figure 1B**). To investigate relationships, mechanisms and functions encoded by differentially expressed genes, NRZ and CRC scores were imported into Ingenuity Pathway Analysis (IPA) (http://www.ingenuity.com/). IPA identified significant activation in the Molecular Mechanisms of Cancer Pathway (**Figure 1C**).

The molecular subtype of subject’s recurrent MB was consistent with the initial diagnosis subtype, SHH, and similarly displayed nodular and desmoplastic histological features. Outside review and cytogenetics showed cytoplasmic expression of GAB1 and beta-catenin, nuclear and cytoplasmic expression of YAP, and lack of widespread overexpression of p53. Genomic findings classified the tumor as being consistent with an SHH-activated pathway and TP53-wildtype MB.

### 3.2 Therapeutic intervention

**Figure 2:**
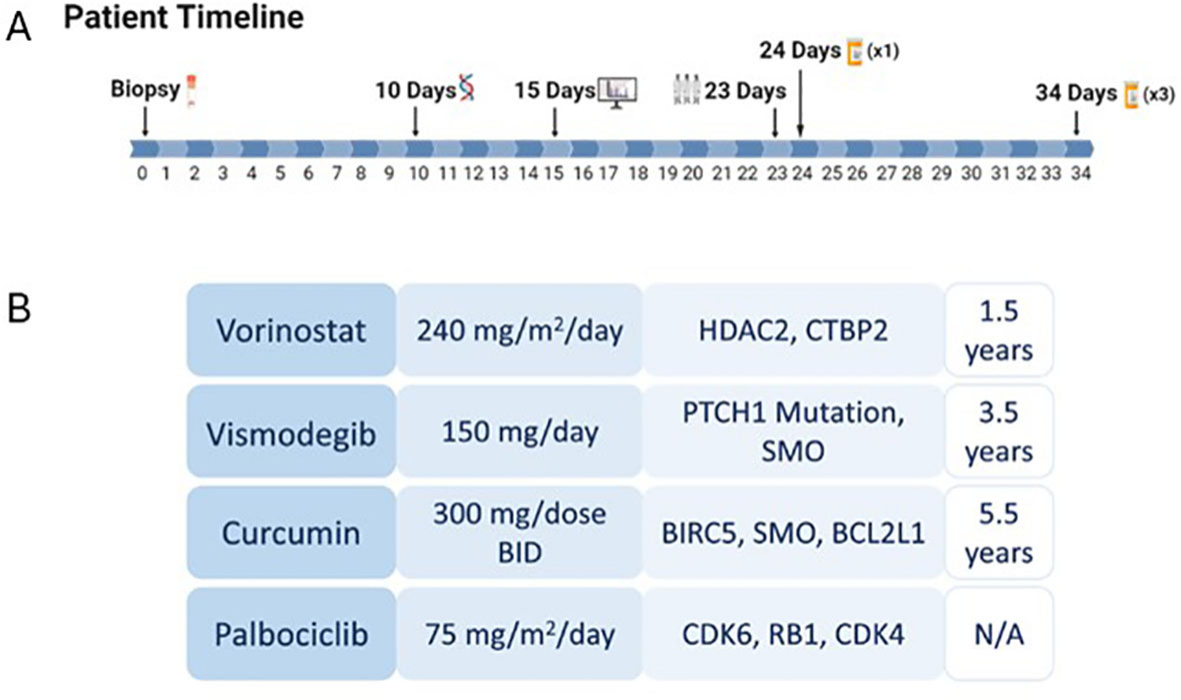
**(A)** Timeline for subject biopsy to sequencing (10 days), genomic analysis (15 days) molecular tumor board (23 days) and start of treatment (24–34 days). **(B)** Molecular tumor board treatment decision with rationale of DNA/RNA targets and time subject received targeted therapy.

A treatment plan was designed by the MTB based on the genomic analysis of this subject’s recurrent MB. The four recommended drugs included vorinostat, vismodegib, curcumin, and palbociclib. Vismodegib was selected due to SMO overexpression (Z-score=1.84; >75^th^ CRC percentile) and is a key part of the SHH pathway. Based on RNA expression analysis, HDAC2 was markedly overexpressed (Z-score=5.55; >75^th^ CRC percentile), as was CTBP2 (Z-score=1.20; >75^th^ CRC percentile), supporting the selection of vorinostat. The drug report further indicated overexpression of BIRC5 (Z-score=3.04), BCL2L1 (Z-score=1.25), and SMO, all of which are known targets of curcumin. Additionally, CDK6 (Z-score=4.67; >65^th >^75th CRC percentile), RB1 (Z-score=3.65; 100^th >^75th CRC percentile), and CDK4 (Z-score=3.07; >65^th^ CRC percentile) were overexpressed, supporting the recommendation of palbociclib. The timeline from biopsy to treatment initiation is detailed in **Figure 2A**. The MTB recommended treatment regimen, dosing and genes of interest are 120 visualized in **Figure 2B**.

MTB reviewed literature regarding these agents in MB (**Figure 1D**). In a Phase I COG trial, two MB subjects had stable disease when given vorinostat alone (20). In a pre-clinical study, administration of the HDAC1/2 selective inhibitor, mocetinostat, to mouse model of SHH-MB caused significant inhibition of tumor growth and prolonged survival (21). Thus, vorinostat was selected based on its ability to target HDAC2 and CTBP2, proven via xenograft to cause significant inhibition of tumor growth and prolonged survival in SHH-MB *in vivo*. In a Phase II Pediatric Brain Tumor Consortium (PBTC) study of adults and children, vismodegib activity was demonstrated in recurrent SHH-MB adult subjects (22). Vismodegib was selected for its ability to inhibit SMO, thereby disrupting activation of the Hedgehog signaling pathway. Curcumin was selected based on gene expression and due to its ability to induce cell cycle arrest and apoptosis in MB cell lines, as well as reduce tumor growth and increase survival in MB xenografts (23, 24). Palbociclib, a CDK4/6 inhibitor, was recommended to target overexpressed cell cycle genes. Pre-clinical activity of palbociclib in MB xenografts has been reported (25). PBTC reported a Phase I clinical trial of palbociclib in children with recurrent, progressive or refractive brain tumors including MB that concluded palbociclib was safely administered (26). However, the subject did not receive palbociclib because he was unable to swallow tablets (due to his age), and a liquid formulation of this drug was not available at that time. Thus, the subject received combination therapy with vorinostat, vismodegib, and curcumin for 1.5 years, as well as craniospinal proton radiation in the first 6 months of therapy.

### 3.3 Follow-up and outcomes

**Figure 3:**
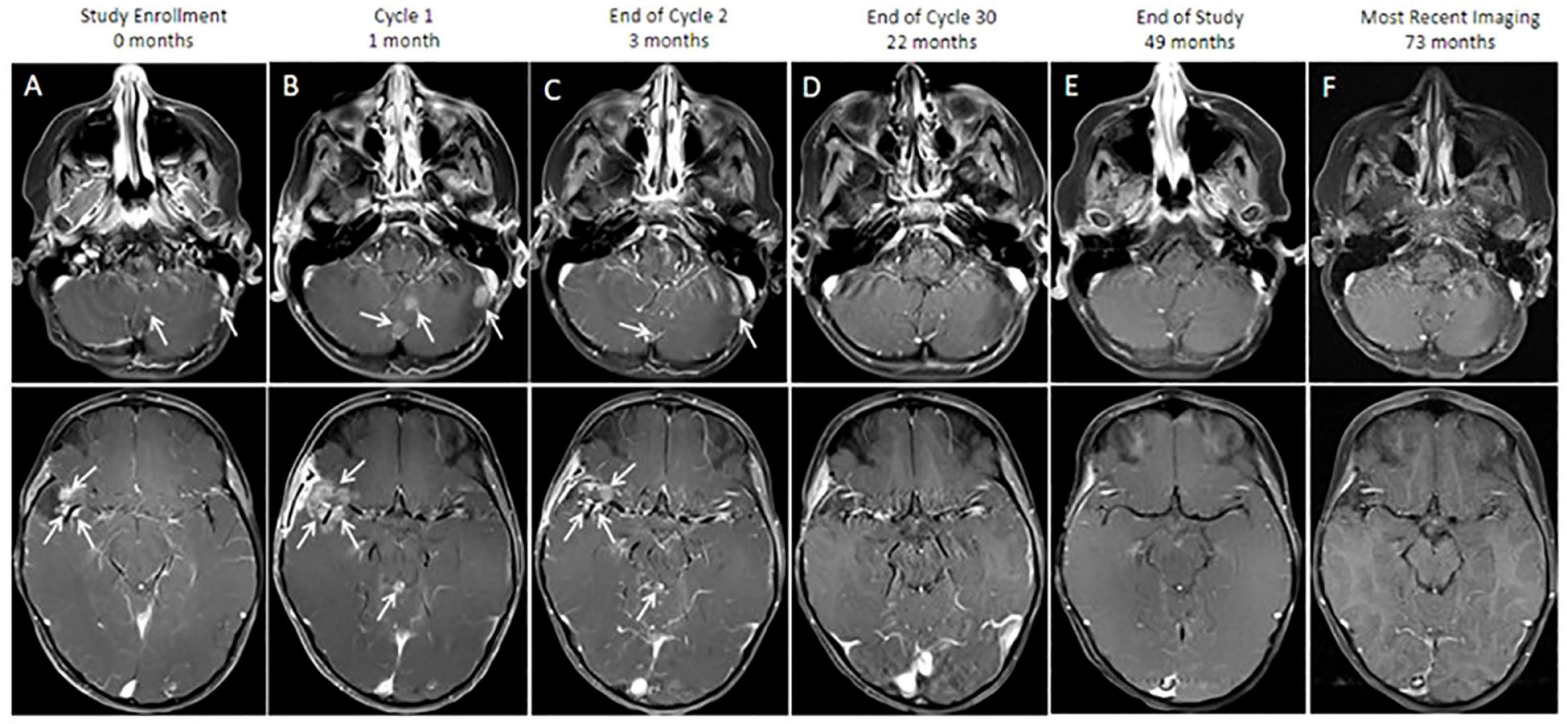
Axial postcontrast T1 TSE images in the upper panel at the level of the posterior fossa and the lower panel obtained at the level of the supratentorial brain show **(A)** At enrollment left cerebellar lateral enhancing nodule (4.6 x 8mm), left cerebellar medial enhancing nodule (4 x 5mm) and irregular enhancing nodule near the right sylvian fissure surgical bed (13 x 18mm). **(B)** Following cycle, one shows enlargement of the left cerebellar nodules (lateral nodule 8x13mm; medial nodule 7.8 x 8.6mm; new posterior nodule 6.6x 7mm). The nodule in the surgical bead near the right sylvian fissure fills most of the postoperative bed and measures 23 x 30mm. A new nodule is seen in the pineal region measuring 7.5x7.7mm. **(C)** At the end of cycle two demonstrates a decrease in size of the enhancing nodules. The lateral cerebellar nodule measures 52.x6.1mm, the medial left cerebellar nodule has resolved and the posterior midline cerebellar nodule measures 2.2 x 3.7mm. The enhancing nodule near the right sylvian fissure measures 8.6x8.6mm with small non-measurable enhancing lesions near the right MCA bifurcation. There is also a decrease in the size of the pineal region enhancing nodule now measuring 3.6x4.8mm. **(D)** At the end of cycle 30, shows complete resolution of all enhancing nodules in the posterior fossa and the supratentorial brain. **(E)** At the end of the study, continues to demonstrate absence of enhancing nodules in the posterior fossa and the supratentorial brain. **(F)** Imaging obtained in 2023 that shows no enhancing nodules. Subject is still without disease as of last imaging May 8, 2025.

In general, the patient tolerated the therapy well and was compliant with the treatment plan. The subject was routinely monitored for side effects and long-term sequelae of this therapy. Of note, vorinostat was discontinued after 1.5 years, due to recurrent mild thrombocytopenia, a known adverse event of this agent. The subject then continued with vismodegib and curcumin for an additional 2 years, and then curcumin alone for another 2 years. Vismodegib was discontinued after an MRI revealed significant growth plate closure, a rare, but known side effect of this agent. The premature growth plate closure was the only Grade 3 or higher adverse event reported for this subject. The treatment regimen duration is visualized in **Figure 2B**. The subject had a complete response after 1.7 years and remains in remission 9 years after treatment initiation (**Figure 3**). With continued long-term follow-up, there have been no additional toxicities. The subject and family perspective was not requested for the purposes of this publication.

### 3.4 Cell line establishment and IC50 values

A cell line was generated from this subject. The tumor was minced and placed in culture media for 15–20 min in a 10cm dish; following incubation all media and tumor fragments were transferred to a T25 flask and cultured following standard practices and maintained in DMEM with 10% FBS, 100U/mL Penicillin, and 100µg/mL Streptomycin.

**Figure 4:**
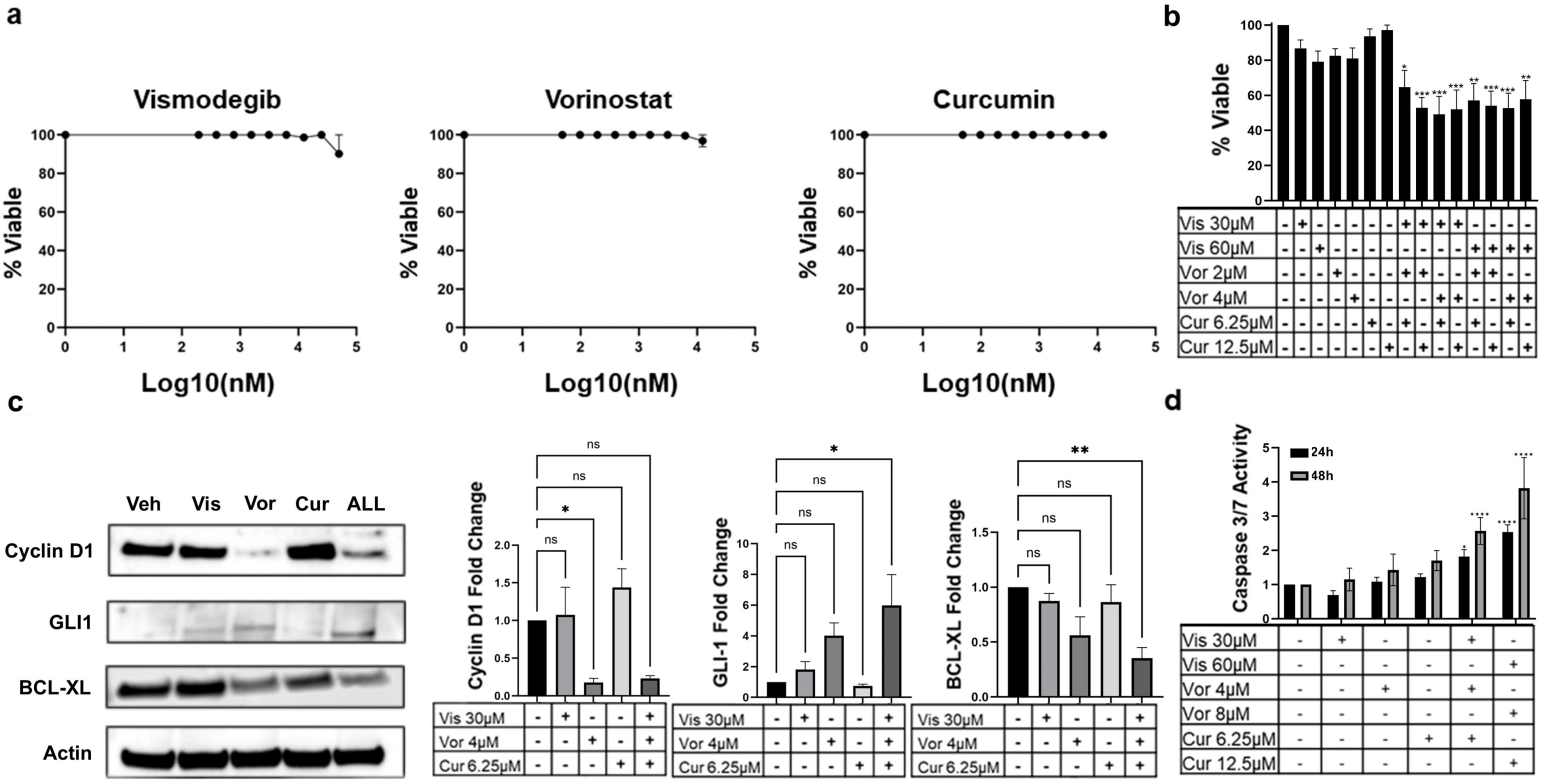
MB subject cells SL00673 were treated for 72 hrs with **(A)** vismodegib (0µM – 60µM), vorinostat (0µM – 50µM), and curcumin (0µM – 12.5µM). **(B)** Combination of vismodegib (30µM), vorinostat (4µM), and curcumin (6.25µM) was then tested for efficacy against each individual treatment and the vehicle-treated cells. Cell viability was measured using CellTiter-Glo; luminescence for each drug treated well was normalized to vehicle treated controls. Data was then graphed and analyzed via ordinary one-way ANOVA using GraphPad Prism (*p = 0.0158, **p < 0.01, ***p < 0.001). **(C)** MB subject cells were treated with the vehicle, vismodegib, vorinostat, curcumin, and combination for 48h and protein expression was determined for the indicated antibodies. **(D)** Additional MB subject cells SL00673 were treated with: Vehicle; 30µM vismodegib; 4µM vorinostat; 6.25µM curcumin; combined drug therapy consisting of 30µM vismodegib, 4µM vorinostat, and 6.25µM curcumin (1x combo); and combined drug therapy consisting of 60µM vismodegib, 8µM vorinostat, and 12.5µM curcumin (2x combo). Cells were collected at 24 and 48 hours and stained for caspase 3/7 cleavage using Cytek Muse Caspase 3/7 Flow Cytometry Kit (#MCH100108), which was then measured using Luminex Guava Muse Cell Analyzer. Data was then graphed and analyzed via 2way ANOVA using GraphPad Prism (*p = 0.0365, ****p < 0.0001).

A total of 3000 cells per well were seeded on a 96 well plate overnight and then treated for 72 hours. Drug concentrations ranged from 0µM – 50µM for vismodegib (Selleck Chemicals), 0µM – 12.5µM for vorinostat (Cayman Chemical Company), and 0µM – 12.5µM for curcumin (Selleck Chemicals). CellTiter-Glo Luminescent Cell Viability Assay (Promega) was used to determine cell viability after treatment; luminescence for each drug treated well was normalized to vehicle-treated controls and GraphPad Prism was used to calculate IC50 values. Each experiment was done in triplicate and replicated for 3 independent experiments. The cell line was resistant to visomodegib, vorinostat and curcumin treatments alone, and therefore IC50s were unable to be obtained (**Figure 4A**).

### 3.5 Combination treatment

Cells were seeded in 96 well plates with 3000 cells per well and incubated overnight followed by treatment with vismodegib (30, 60µM), vorinostat (2, 4µM), and curcumin (6.25, 12.5µM). Two treatment dosages were used for single and combination treatment, with lower dosages around Cmax and higher dosages doubled as 50% inhibition was not observed in any single agent. CellTiter-Glo Luminescent Cell Viability Assay (Promega) was used to assess cell viability following the manufacturer protocol. Each experiment was performed in triplicate and replicated for 3 independent experiments. GraphPad Prism was used for statistical analysis. Combination treatment was effective, with Vis30 + Vor4 + Cur6.25 demonstrating the greatest efficacy and reducing cell viability to 40% (**Figure 4B**).

### 3.6 Western blot analysis

180,000 cells were seeded per well in a 6 well plate overnight and treated for 48 hours. Cells were treated with the vehicle, vismodegib, vorinostat, curcumin, and combination (30uM vismodegib, 4uM vorinostat, 6.25uM curcumin). Cells were lysed in RIPA buffer containing protease and phosphatase inhibitors (Thermo Scientific). Protein concentrations were determined by Pierce™ BCA Protein Assay Kit (Thermo Scientific). Lysates were denatured at 95 °C for 5 minutes and subjected to electrophoresis on a 4-12% Bis-Tris gel (25µg of protein were loaded per lane). Gels were transferred to a nitrocellulose membrane using the iBlot 2 Dry blotting system (Invitrogen). Protein expression was detected using the Radiance Plus or Radiance Q chemiluminescent substrate (Azure Biosystems) and imaged using the Azure 600 Imaging System (Azure Biosystems). Primary antibodies used were: rabbit polyclonal ß-Actin (#4967), GLI-1 rabbit monoclonal (#3538), BCL-XL rabbit monoclonal (#2764) from Cell Signaling, and mouse monoclonal Cyclin D1 (05-362) (Millipore Sigma). Secondary antibodies used were: HRP-linked anti-rabbit IgG (#7074) and HRP-linked anti-mouse IgG (#7076) (Cell Signaling). Western blot analysis showed that vorinostat decreased cyclin D1 and BCL-XL expression while increasing GLI-1 levels; these effects were further enhanced with combination treatment. (**Figure 4C**).

### 3.7 Caspase 3/7 activity assay

180,000 cells were seeded per well in a 6 well plate overnight and treated for 24 and 48 hours. Cells were treated with the vehicle, vismodegib, vorinostat, curcumin, 1x combination (30uM vismodegib, 4uM vorinostat, 6.25uM curcumin), and 2x combination (60uM vismodegib, 8uM vorinostat, 12.5uM curcumin). Cells were then trypsinized and stained using Cytek Biosciences Muse Caspase 3/7 Activity Kit (item number MCH100108), which was then quantified using Luminex Guava Muse Cell Analyzer. Assay was performed in triplicate according to manufacturer instructions. Caspase 3/7 activity was modestly increased with each individual agent but was significantly elevated with combination treatment (**Figure 4D**).

## 4 Discussion

Overall, multi-modality therapy with surgery, chemotherapy, and radiation has improved survival rates in pediatric patients with MB. Prognosis is affected by factors such as tumor grade and other molecular characteristics, subject age and health status at diagnosis, and response to therapy. Survival rates for localized MB are approximately 72.1% after five years and decrease to about 60% for metastatic disease to the spinal cord or cerebrospinal fluid. In addition, many of these patients suffer significant long-term sequelae. Approximately 30% of all patients experience relapse, with survival rates less than 5% (1, 3). Treatment after relapse can include chemotherapy, radiation, and other symptom-modifying care (27). Even if treatment after relapse results in a successful outcome, many of these patients suffer from severe long-term sequelae.

For patients who did not receive radiation at diagnosis, radiation at relapse does improve EFS (3) and likely played a role for this subject. Most patients also require additional chemotherapy to prevent further relapse or progression. This study suggests that genomic sequencing was able to be used in an MTB to identify therapeutic options for this subject. Specifically, this subject was treated safely with the novel combination of vorinostat, vismodegib, and curcumin without significant unexpected adverse events during therapy. A known long-term toxicity associated with vismodegib is premature fusion of epiphyses which occurred 3 years after starting this medication. Nine years post relapse, this precision medicine approach was associated with a sustained tumor response and long-term survival.

A patient-derived cell line was successfully established for this subject. Combination therapy was required for *in vitro* response in the patient-derived cell line, as the single agents alone were ineffective in decreasing cell viability, but the combination resulted in decreased cell viability. IC50 curves were generated for each single agent treatment (**Figure 4A**) with minimum dosages being no treatment and maximum dosages being: 60µM for vismodegib (cmax 33.9µM) and vorinostat (cmax 1.2µM) as well as 12.5µM for curcumin (cmax 6.3µM). These IC50 curves, showing a lack of efficacy of single agents, clearly demonstrate the necessity of the three-drug combination. Furthermore, western blotting showed that expression of cyclin D1 and BCL-XL is decreased by combination treatment, suggesting a decrease in cell cycling and increase in apoptosis. This proposed increase in apoptosis is corroborated by the increased concentrations of cleaved caspase 3/7 via flow cytometry (**Figure 4D**). Overall, these results suggest that combination therapies may be needed to overcome resistance. This correlates with outcomes seen in early phase single-agent-versus-combination drug studies where response rates are improved in combination trials (28).

BCC clinical trial, NMTRC009, looked at the feasibility of providing molecular-guided therapy (MGT) for the treatment of patients with relapsed/refractory childhood cancers. The trial evaluated 144 subjects for safety and 124 subjects for response. The average number of days from biopsy to initiation of the MTB-recommended combination therapy was 38 days. Patient benefit was exhibited in 65% of all subjects: 67% of neuroblastomas, 73% of CNS tumors, and 60% of rare tumors (14). There was little associated toxicity above that expected for the MGT drugs used during this trial, suggestive of the safety of utilizing this method of selecting novel combinations of targeted therapy.

Genomic analysis and MTB are used more commonly to identify therapies for patients. MTBs can help clinicians navigate the complex world of precision medicine and provide advanced treatments tailored to their patients’ specific genomic sequencing results. MTBs include a wide range of professions to guide the treatment recommendations including clinical oncologists, pharmacists, genomic experts, bioinformaticians, and geneticists. There have been several studies conducted evaluating the feasibility and efficacy of utilizing MTBs and WES/WTS sequencing to create individualized treatment plans for difficult to treat tumors (29–32). Berger et. al., found that in tumors with a high mutational load, data from RNA sequencing can help to identify the mutated genes that are driving tumor progression or, more generally, can be interpreted to evaluate the biology of the tumor itself in terms of the cancer subtype, aggressiveness, potential to metastasize, and other biological aspects (33). Despite these promising results, limitations of this approach include it being a single patient experience, challenges in interpreting and prioritizing clinically actionable findings from large, complex genomic datasets and in obtaining timely insurance approval for off−label targeted therapies. A key limitation of this study is that the patient-derived cell lines can undergo phenotypic and molecular drift over time due to the absence of a native tumor microenvironment and the selective pressures imposed by *in vitro* culture media. Consequently, such models may not fully recapitulate the exact molecular state of the patient’s tumor at the time of sampling. As a future direction, we aim to establish organoid models and 3D culture systems that better emulate the tumor microenvironment and preserve patient-specific cellular interactions.

In conclusion, the application of complex genomic sequencing, specifically RNA, in treating this case of pediatric medulloblastoma has demonstrated the potential of precision medicine to significantly improve treatment outcomes. By identifying specific genetic mutations and pathways, we were able to tailor a combination of up to four targeted drugs that effectively addressed the unique molecular profile of the tumor that single agent therapy is unable to accomplish. This personalized approach not only resulted in a successful therapeutic response but also minimized adverse effects, highlighting the importance of integrating genomic data into clinical decision-making. Our findings underscore the promise of RNA sequencing as a powerful tool in the fight against pediatric cancers, paving the way for more individualized and effective treatment strategies.

## Data Availability

The datasets presented in this study can be found in online repositories. The names of the repository/repositories and accession number(s) can be found below: https://www.ncbi.nlm.nih.gov/gap/, phs002238.v1.p1.
